# Small RNA Activity in Archeological Barley Shows Novel Germination Inhibition in Response to Environment

**DOI:** 10.1093/molbev/msx175

**Published:** 2017-06-24

**Authors:** Oliver Smith, Sarah A. Palmer, Alan J. Clapham, Pamela Rose, Yuan Liu, Jun Wang, Robin G. Allaby

**Affiliations:** 1 School of Life Sciences, The University of Warwick, Coventry, United Kingdom; 2 The Austrian Archaeological Institute, Cairo Branch, Zamalek, Cairo, Egypt; 3 BGI-Europe-UK, London, United Kingdom; 4 BGI-Shenzhen, Shenzhen, China

**Keywords:** ancient RNA, miRNA, adaptation

## Abstract

The recovery of ancient RNA from archeological material could enable the direct study of microevolutionary processes. Small RNAs are a rich source of information because their small size is compatible with biomolecular preservation, and their roles in gene regulation make them likely foci of evolutionary change. We present here the small RNA fraction from a sample of archeological barley generated using high-throughput sequencing that has previously been associated with localized adaptation to drought. Its microRNA profile is broadly similar to 19 globally distributed modern barley samples with the exception of three microRNAs (miRNA159, miRNA319, and miR396), all of which are known to have variable expression under stress conditions. We also found retrotransposon activity to be significantly reduced in the archeological barley compared with the controls, where one would expect the opposite under stress conditions. We suggest that the archeological barley’s conflicting stress signals could be the result of long-term adaptation to its local environment.

## Introduction

As domesticated plants were transported from their areas of origin they continued to evolve and adapt under domestication to these new environments. Such adaptations included to changes in seasonality (Yan etal. 2004, 2006; Dubcovsky etal. 2005; Fu etal. 2005) and to local conditions such as water stress (Araus etal. 2014). Understanding how plants became adapted to local environments is important for understanding the domestication process, and how food security issues of the past were met. Archeological barley from the site of Qasr Ibrim, situated in the upper reaches of the Nile, is interesting because it has been identified as a crop that appears to have adapted to local water stress conditions on the basis of archaeogenetic evidence (Palmer etal. 2009; Allaby etal. 2015). The crop was grown by successive cultures from the Napatan, to the Islamic over a period of 3,000 years ending a few hundred years ago.

Barley spikelet architecture generally takes one of two forms, or intermediates: two-rowed and six-rowed. Rowedness determines the number of grains per rachis node and is controlled primarily by VRS1, a transcription factor involved in suppressing lateral spikelet formation (Komatsuda etal. 2007). In many domesticated varieties, a loss-of-function mutation in the VRS1 gene (a deletion resulting in frame shift) renders the resulting *vrs1.a1* allele inactive, resulting in development of spikelets lateral to the two central spikelets and hence six rows of grain. In all cultural stages, the barley at Qasr Ibrim shows a two-row phenotype combined with the genetic basis of a six-row type. We confirmed the genetic basis of the six-rowed type in the Qasr Ibrim barley by sequencing the *vrs1* (Palmer etal. 2009) and *int-c* loci (this study, see Materials and Methods) and found both to be consistent with the respective six-rowed alleles. This suggests a reassertion of two-row over six-row through a novel mechanism that is indicative of a strong selection pressure. The most likely pressure in this case was water stress given the aridity of the site and extremely short growing season determined by basin irrigation (Palmer etal. 2009). It is therefore interesting to establish the genomic basis of stress tolerance associated with this crop.

Archaeogenomic approaches provide a means to directly observe genomic change in the past, and have demonstrated that domesticated plants can show considerable change under domestication that could relate to local adaptation (Palmer etal. 2012). Alternatively, ancient RNA, rather than DNA, could provide a means of direct observation of phenotypes of selective value that would otherwise have been difficult to predict accurately from DNA sequence data alone. There have been few studies on ancient RNA because of concerns about its high rate of degradation (Lindahl 1996; Soukup and Breaker 1999), especially from hydrolytic attack (Thompson etal. 1995). However, under arid conditions RNA is likely to be more stable over time (Fordyce etal. 2013; Smith etal. 2014a). In particular, the small RNA fraction is of potential interest for ancient biomolecule studies of evolution because their small size (18–24 nucleotides) is about half the typical median size of ancient DNA fragments (Palmer etal. 2012), and their recovery from ancient, preserved tissues, with expected tissue specificity, has recently been demonstrated (Keller etal. 2016).

Small regulatory RNAs are a major component of gene regulation networks acting through various mechanisms including histone modifications (Gonzalez etal. 2008), DNA methylation (Li etal. 2008), translational inhibition (Baulcombe 2005) and transcription silencing (Guo etal. 2005). As such, small RNAs are critical mediators of regulatory activity in plant growth, development and responses to biotic and environmental stresses (Talame etal. 2007; Kantar etal. 2010; Yao etal. 2010; Khraiwesh etal. 2012). The expression levels of small RNAs can vary significantly under various stresses and at different developmental stages (Bartel and Bartel 2003; Achard etal. 2004). One class of small RNA, microRNA (miRNA), are formed from conserved biogenetic pathways in which a primary microRNA transcript (pri-miRNA) forms a precursor stem loop structure (premiRNA) which is cleaved by a DICER-like enzyme (DCL) to produce a precise size class of miRNA molecule of 21–24 nucleotides, depending on the pathway and specific DCL involved. The miRNA is then exposed by removal of its complementary passenger strand (miRNA*) and mediated to its target through an AGO/RISC complex (Bonnet etal. 2006). MicroRNAs can be readily identified due to their highly conserved nature among flowering plants, their characteristic stem loop structures that are formed during processing (Sunkar and Zhu 2004; Axtell and Bartel 2005), and that they are often involved in highly conserved gene regulatory networks. A second class of small RNA, short interfering RNA (siRNA), are significantly less conserved between species than miRNAs and carry out a broader range of functions. The biogenetic pathway of siRNAs does not involve stem loop formation, and most mRNA have the potential to become a source of siRNAs (Carthew and Sontheimer 2009). Consequently, siRNAs are harder to predict in silico than miRNAs, but may evolve more rapidly.

To investigate the possible regulatory basis of stress tolerance in the archeological barley from Qasr Ibrim, we isolated the small RNA fraction of the transcriptome and compared it to the small RNA fraction of 19 modern, globally distributed barley seeds. The distribution included samples from Africa, the Near East, South America, Japan, and Europe.

## Results and Discussion

### RNA Content of Archeological Seeds

On the basis of six desiccated grains dated to the Late Christian period (600–900 years BP; contextual dating), the total RNA content of the archeological barley was determined to be three times that of the DNA content with an average of 1.45 µg per seed DNA content and 4.45 µg per seed RNA content. A total of 59,702,667 reads were generated from the archeological barley using the Illumina HiSeq 2500 platform, and an average of 696,171 reads were generated from each of the control samples using the Illumina MiSeq platform. We opted for a single-sample run using the HiSeq for the archeological sample to preempt low copy numbers of microRNA as a result of diagenesis, and to compensate for possible overrepresentation of other RNA breakdown products. Of the archeological reads generated, 59% fell into the small regulatory RNA size range, whereas the same average figure was 17% for the modern barley. Metagenomic analysis established that 93% of assignable reads were barley in the archeological data set (supplementary fig. S1, Supplementary Material online), which is comparable to previous analyses with cotton from the same site. To verify this, we performed a modified version of Phylogenetic Intersect Analysis pipeline (Smith etal. 2015) on a subset of reads, and found the endogenous content to be at least 87.6% (see Materials and Methods). Human contamination was found to account for only 0.01% of the assignable reads, indicating the acceptably low level of background contamination. The gross measurement of RNA and DNA content is therefore a close reflection of the true endogenous content, and indicates that RNA degradation is greatly slowed down in arid environments. A previous study calculated that at the Qasr Ibrim site ancient DNA has a half-life of 350 years (Palmer etal. 2009), and we calculated the equivalent RNA half-like to be between 155 and 232 years. These results support a 1.5–2.3 fold higher rate of degradation of RNA than DNA if one assumes equal relative quantities of total RNA and DNA in archeological and modern tissue, indicating that the arid preservation conditions of Qasr Ibrim decrease the relative rate at which RNA degrades compared with DNA from the 50-fold difference observed by invitro studies (Eigner etal. 1961).

The composition of the barley small RNA was similar between control samples, but the archeological sample was markedly different. The majority of reads from the archeological sample were assigned as ribosomal at 71% compared with a mean of 23% in the control samples. tRNA and mRNA were all likewise highly overrepresented in the archeological sample proportionally to miRNA, compared with all control samples (supplementary fig. S2, Supplementary Material online). This is likely a result of RNA fragmentation during diagenesis, causing a greater proportion of breakdown products falling into the size range being studied.

### Profiles of miRNAs in Archeological and Control Barleys

1938 HiSeq reads from a total of 59,702,667 reads were identified as known miRNAs from the archeological barley, representing 23 families referenced against miRBase (Griffiths-Jones etal. 2008). 22053 MiSeq reads from a total of 13,227,250 representing 36 families were identified from the control samples, as expected an order of magnitude greater than the archeological sample (supplementary table S1, Supplementary Material online).

While the profiles between control samples show high levels of similarity (correlation regression matrix; all *P* values <0.0001), the profiles of archeological and control samples however differ significantly; linear regression shows a low degree of correlation (*R*^2^ = 0.037, *P* = 0.3). This is due to notable differences in four miRNA families: miR159, miR319, and miR5139 both highly abundant in the archeological sample compared with the controls, and miR396, highly abundant in the control samples compared with the archeological sample (supplementary fig. S3, Supplementary Material online). Removal of these microRNA families from statistical analysis shows that the baseline miRNA activity is similar between all samples (regression *R*^2^ = 0.724, *P* = < 0.0001). The similarity of these profiles suggests that these two data sets can be directly compared meaningfully, and supports the notion that RNA frequencies can be usefully retrieved from the archeological record.

The most notable difference between the modern and archeological profiles was that of miR396, substantially downregulated in the archeological barley. To detect more significant variations between the two profiles we calculated the posterior distributions of the underlying λ values giving rise to the counts observed, assuming an extended Poisson process in which the mean to variance ratio was calculated from the average of each of the pairwise counts in the two profiles (Faddy 1997), see Materials and Methods. In the cases of miR159 and miR319, we detected an upregulation in the archeological barley and downregulation of miR396 (supplementary fig. S4, Supplementary Material online). The presence of stable expressions of three stress-related miRNAs in a range modern barleys suggests a global uniformity under unremarkable conditions, and a bimodal expression pattern in miR396 among the modern barleys further suggests two stable expression states. Conversely, the Qasr Ibrim barley significantly deviates from the modern stable expression patterns. This, coupled with phenotypic differentiation but little evidence of biomass loss, such as diminished seed size, suggests the possibility that this barley has alternate expression patterns which were an adaptation to the environment, rather than immediate stress response.

### The Effects of Divergent miRNA Profiles on Barley Physiology

The primary targets of the most upregulated microRNA, miR396, are growth-regulating factor (GRF) genes (Liu etal. 2008; Debernardi etal. 2012). GRF control seed development and plant architecture and their down-regulation by miR396 in plant tissues results in growth inhibition (Rodriguez etal. 2010) and there is evidence of their association with dormancy (Martin etal. 2006). Conversely, down-regulation of miR396 itself is associated with drought stress response in rice (Zhou etal. 2010). Seemingly key to its effect is the plant’s developmental stage. Extended expression during growth suppresses cell division (Rodriguez etal. 2015) whereas in mature plants, targeted blocking of it increases rice yield by allowing GRF6 expression (Gao etal. 2015). Overexpression of miR396 in rice under otherwise unstressed conditions gives rise to abnormal floret development and suggests a complex regulatory network (Liu etal. 2014). The persistent, arid paleoclimate of lower Nubia combined with the obvious difference in profiles, particularly the down-regulation of miR396 in the archeological barley, likely suggests a stress response.miRNAs 319 and 159 are known to share similar transcript targeting function (Li etal. 2011), and surprisingly, similar regulatory feedback mechanisms (Reichel and Millar 2015). miR319 in particular is well-known as a stress-induced regulator in agronomic plants (Zhou and Luo 2014) and miR159 is highly conserved across plants as well as being equally known for stress responses (Li etal. 2016). The primary targets of miR319 are class II TCP domain containing proteins (Schommer etal. 2008), in particular PCF5, PCF6 and GAMYB (Li etal. 2011; Thiebaut etal. 2012). To distinguish between these possibilities, we searched the messenger RNA fraction for fragments of these targets. Only trace quantities of PCF5, PCF6, and GAMYB were detected in 7 of the 19 control samples with equal representation, but in the archeological barley PCF transcripts were in more abundance than GAMYB transcripts by an order of magnitude. Analysis of the posterior distribution of GAMYB counts showed that the archeological sample had fewer fragments than modern barleys, consistent with a more stringent posttranscriptional silencing in the archeological sample (supplementary fig. S5, Supplementary Material online). This result makes sense, because GAMYB is involved with the mobilization of the aleurone layer during germination, whereas PCF5 and PCF6 are involved in later stages of embryogenic development (Gubler etal. 1999; Kaneko etal. 2004; Thiebaut etal. 2012). GAMYB is also the target of miR159, which is the most highly expressed miRNA in the archeological sample. In the case of the archeological barley it seems that two miRNA loci have assumed this function in fairly equal measure. The added action of miR319 is likely to have the effect of delaying the onset of germination. In this case, the extreme difference in count is suggestive to us that the archeological barley is responding to stress via an alternative pathway, by preventing germination via miR319 and miR159 action where miR396 is less available due to another facet of stress response. This unusual response mechanism may be due to this particular cultivar being locally adapted to the conditions at Qasr Ibrim.

### GC Content of Archeological RNA

Keller etal. (2016) found a small but present correlation between GC content and expression levels of mammalian microRNAs in permafrost remains using miRNA-specific qPCR, suggesting a preferential long-term survival rate for fragments with higher GC content. We compared the average GC content of identified plant miRNAs in our samples to the relative expression levels of each, and found a weak positive correlation in both (*r*^2^ = 0.00059, in the ancient, and 0.00541 in the modern, supplementary fig. S6, Supplementary Material online). The fact that a stronger correlation exists in the modern samples suggests that any preferential survival of GC-rich molecules in the archeological sample is not a significant issue. Further, the three most differentially expressed miRNAs do not deviate significantly from the median value of the GC distribution (54.2%) and the two that are highly expressed in the archeological sample, miR159 and miR319, fall at 47.6% and 57.1%, respectively. We also note that the mean GC content of miRNA in the archeological barley (54.7%) is higher than that of the controls (53.0%), leading us to surmise that although a preferential survival of high-GC molecules is still present, the expression correlation in this case is rendered negligible by the miRNA expression profile differences between modern and archeological samples. Other factors such as tissue type, burial conditions, and the method of data generation, which are quite different between our samples and the Tyrolean iceman, may also be at work, although a current lack of data concerning archeological miRNAs makes further speculation difficult.

### Retroelement Activity in Archeological Barley

To determine whether it is more likely that the miRNA expression profiles in the archeological barley represent an immediate stress response to the environment or a more stable adaptive state, we assessed the inferred state of stress by examining evidence of retroelement activity. Increased retroelement activity under a variety of biotic and abiotic stress in plants has been well documented (Wessler 1996; Grandbastien 1998; Bui and Grandbastien 2012). A total of 57,650 reads were identified in the archeological sample as being retroelement-targeting siRNA or retroelement transcript breakdown products. 23,313 reads in total were identified as such from the controls, with a mean frequency of 1,227 (standard deviation 348). Adjusting for read frequency differences between multiplexed controls and expressing as a per-sample proportion, retroelement-originating reads accounted for 0.24% of all RNA species in the archeological sample, and in the modern samples, between 0.48% and 1.78% of the total small RNA (sRNA)-sized reads with an average of 1.12% (standard deviation 0.0038). Proportions of retroelement-originating reads were therefore 4.6 times higher in the control samples but in a similar order of magnitude to the archeological sample. We then calculated the proportions of retroelement transcripts to messenger RNA only, excluding ribosomal RNA sequences, since responsive rRNA expression is not well characterized and should not be assumed to reflect environmental stress. In this case, retroelement transcripts accounted for 0.69% of the archeological mRNA, and between 0.67% and 2.61% in the controls, with a mean value of 1.72% (standard deviation 0.0065).

These data suggest retroelement activity in the archeological sample is on a comparable order of magnitude to the controls, often lower, and therefore not indicative of an increased stress response relative to the controls. Previous work relating to the same sample (Smith etal. 2014b) has shown decresed retroelement expression in conjunction with increased methylation around known retroelement-containing loci. The elevated methylation patterns are likely to be a result of biotic infection noted in the sample at that stratum only, and the expectation of global genomic methylation under infections would include methylation of retroelement-containing loci, and so not be symptomatic of retroelement copy number variation. Genomic rearrangement of transposable elements in plant evolution is well documented (Palmer etal. 2012), although assessing copy number from a fragmented genome is highly error-prone. We therefore conclude that these data are congruent with our previous interpretation of normal or reduced retroelement activity.

## Conclusions

The unusual two-row phenotype of the barley at Qasr Ibrim could have been formed by either a novel genetic mechanism that reasserted inhibition of the lateral florets from a six-row ancestor, or it could have been due to widespread floret failure caused by environmentally induced stress. The findings in this study of an unusual microRNA profile suggest a stress response, as do previous data of high levels of genomic methylation around retrotransposon-rich loci (Smith etal. 2014b). Our observations of normal retrotransposon activity seemingly do not support this (supplementary table S2, Supplementary Material online). However, studies of epigenetic transgenerationalism show retroelement activity in response to stress is not necessarily continuous (Ito etal. 2011). This suggests that the Qasr Ibrim barley’s unique profile could be the result of localized adaptation to environmental stress, possibly over an extended period, resulting in the phenotype and genotype observed in a previous study (Palmer etal. 2009). Local adaptation is difficult to establish in the archeological record. However, the consistency in the *vrs1* allele diverging from the expected phenotype at multiple strata observed at Qasr Ibrim, combined with all other similar studies showing an expected match between phenotype and *vrs1* allele (Mascher etal. 2016; Hagenblad etal. 2017), suggests that local adaptation is indeed present in this case.

The regulatory profiles detected in this study mostly concerned the regulation of germination and showed that distinctly different mechanisms are present between archeological and modern barleys. The archeological barley was significantly more inhibited by miRNA319 and miR159 that would likely have the cause of preventing germination in a mechanism unique to this cultivar. In the case of the modern counterparts, miR396 appears to take this role; in fact, stress-induced down-regulation of miR396 in the archeological barley could have resulted in the alternative mechanism seen in the archeological barley, which over several generations has become stabilized as an adaptation. This regulatory difference makes sense given the basin irrigation system in which the archeological barley was grown. In ancient Nubia the length of the growing season (*Peret*) was determined by the Nile flood and the ensuing time it took for the soil to dry out again, about 120 days (Kemp 2006). The length of this season corresponds very closely to the length of time required for barley to grow. Consequently mechanisms that prevent early onset of germination, before the floods, may well have been important to avoid a high mortality of seedlings.

This study is the second to recover ancient small regulatory RNA and shows that it can be meaningfully used to contrast how ancient organisms fared in their environment relative to their modern counterparts. We suggest that the evidence presented here shows that the archeological barley was adapted through stress responses to the agrarian environment around the Nile as exemplified through its differential regulation of germination and floral structure. Such examples of local adaptation highlight the increasingly probable notion that epigenetic regulation is a driver for evolution, and the possibility that some issues of food security in the past may have been better addressed than in the present.

## Materials and Methods

### Samples

Archeological barley from Qasr Ibrim from the Late Christian horizon (1100–1400 AD) was supplied by Dr Clapham. 15 accessions of modern barley were supplied by the USDA: PI 642783 (Giza), PI 182613 (Japan), PI 392528 (South Africa), PI 510559 (Peru), PI 524707 (Egypt), PI290743 (Kenya), PI 100121 (India), PI 65216 (China), PI 524708 (Egypt dryland), PI 168904 (Argentina), PI 490393 (Mali), PI 642787 (Giza), CIho5059 (Australia), and GSHO842 (Japan). Two accessions were supplied by the John Innes Centre: B9604 (Iraq) and B7297 (Iran). Two accessions were supplied by NIAB: Tipple and Line 14 hulled (both U.K.).

### Nucleic Acid Extraction

All ancient nucleic acids were handled in a dedicated ancient DNA laboratory. RNA was isolated using a modified protocol of the Ambion MirVana RNA isolation kit. Six grains from the same archeological context were extracted in bulk as a single sample. Modifications included an extended incubation time in 2% CTAB buffer (5 days at 37°C) followed by a single chloroform extraction and isopropanol precipitation, before resuspending the RNA pellet in the supplied binding buffer and continuing the protocol as per the manufacturer’s instructions. Because the vast majority of archeological RNA fragments already exist within the target size for column based enrichment of small fragments (<300 nt), this step was deemed unnecessary and omitted to avoid further loss of molecules. RNA was extracted from control samples in a separate, nonPCR laboratory using a modified protocol of the Ambion MirVana RNA isolation kit. Crushed seeds were incubated for 1 h at 65°C in 2% CTAB buffer followed by a single chloroform extraction and ispropanol precipitation. Due to the high starch content of modern grains compared with the archeological, RNA pellets were dissolved in SSTE buffer to remove polysaccharides, and RNA reprecipitated with isopropanol. Pellets were resuspended in the supplied binding buffer and the protocol was completed according to manufacturer’s instructions. Small RNA fragments (<300 nt) were enriched using the manufacturer’s column-based method. Since mechanical size enrichment of this type has not been reported to introduce sequence bias, we do not anticipate this modification influenced the results. Further, we do not see any order-of-magnitude differences in reported relative expression from similarly sized transcripts such as transposable elements or ribosomal transcripts, suggesting the differences in protocol introduce minimal, if any, bias into our molecular classification. RNA for all samples were quantified using the Qubit RNA assays.

### Illumina Library Construction and Sequencing

A singleplex library was created for the archeological sample using an Air Small RNA Library Preparation Kit (Bioo Scientific, cat. no 5130, discontinued) according to manufacturer’s instructions, and run as a single lane on the Illumina HiSeq platform by BGI (Shenzen, China). Control sample libraries were made using a NextFlex Small RNA Sequncing Kit (Bioo Scientific, cat. no. 5132-06) due to multiplexing allowances, again following the manufacturer’s protocol. We used standard indexing primers for multiplexing, and run on the Illumina MiSeq platform using a v2 reagent kit. All library enrichment took place using eight PCR cycles, and all sequencing runs took place with 50 rounds of sequencing.

### Bioinformatic Methods

Primary data processing (format conversion, removal of adapter sequences, parsing into 18–25 sRNA-sized sequences and identification of sequence frequencies in reads) was carried out using scripts of the author’s design. The majority of pattern matching exercises were performed using bowtie version 0.12. Separate bowtie indexes were created for each molecule type from corresponding fasta files: complete barley genome cultivar Morex, mature plant miRNA from mirbase, complete graminae retroelements from Michigan State University’s Plant Repeat Database, and individual genes of interest (PCF5, PCF6 and GaMyb) from NCBI. In all cases of bowtie pattern matching, a single mismatch was allowed in the archeological sample but only where a positive-strand C > U or negative strand G > A base modification occurred. This was to compensate for postmortem deamination known to occur in ancient nucleic acids (Gilbert etal. 2005). For the control samples, exact matches only were considered positive.

No comprehensive list of primary miRNA transcripts exists, so a database was created based on homologous premiRNA stem loop sequences. Pri-miRNA sequence data for all available plant species was downloaded from mirbase and subjected to BLAST searching against a database created from the barley cultivar Morex genome (contigs of ∼100–800 nt each). PremiRNA sequences matching to the Morex genome under standalone BLASTn parameters were considered to be homologous if originated in other plant species. Morex contigs to which homologous stem-loop sequences matched were considered to be representative of miRNA primary transcripts. These contigs were extracted and converted to a bowtie index to which the Illumina sequences of archeological and control samples were matched, again allowing a single deamination mismatch in the archeological sample.

For metagenomic analysis, sequences were processed using standalone blastn, using the complete nucleotide (nt) database and preset parameters for short sequences (“blastn-short”), which reduces word size values to 7 over the default 11. This was deemed the most appropriate method due to the ultrashort nature of sequences being evaluated.

Phylogenetic Intersect Analysis (PIA) was performed on a subset of reads to verify the expected levels of endogenous miRNAs, by proxy of confidence in their taxonomic assignations. Plant small RNA fractions are typically ultrashort and highly conserved, and this poses challenges for discriminant phylogenetic analyses such as PIA. Essentially, many sRNA sequences are so highly conserved, and by virtue of their sizes are often coincidentally present in nonplants, they are unresolvable from almost a basal level on the phylogenetic tree. To account for this, we took 10,000 redundant sequences and ran them through the PIA pipeline as normal. We disregarded those sequences whose second BLAST hit had an identical score to the first, then filtered the remaining reads by disregarding those with an unknowable classification intersect value. We then remapped the remaining reads to the barley genome. Finally, we assessed the number of exogenous reads by isolating those meeting two criteria: not mapping to the genome, and not consistent with a putative barley assignation under PIA (i.e., not being assigned at any taxonomic node leading to barley). We then applied redundancy frequencies to these data to calculate the overall proportions of endogenous and exogenous reads. We found the endogenous content of the barley to be at least 87.6%, and the accuracy of PIA to be 80.6%, both values being consistent with our previous estimates (Palmer etal. 2012; Smith etal. 2015).

### *Int-c* Alleles of Qasr Ibrim Barley

Barley architecture is controlled by both *VRS1* and *INT-C* genes (Ramsay etal. 2011). To check whether the two-row phenotype of the Qasr Ibrim barley could be accounted for by a combination of *vrs1* and *int-c* alleles which have been known to be responsible for a two-row phentotype (Ramsay etal. 2011) despite the presence of the 6-row associated *vrs1.a1* allele. We mapped 6,731 Illumina genomic DNA reads generated from seeds of the same archeological deposit as the miRNA-associated sample to all five alleles of the *int-c* locus, using bowtie2. A single *int-c* genotype (*Int-c.a*; Genbank accession KY070602) was found in the Qasr Ibrim barley across samples, which is the *vrs1* allele associated with the six row phenotype.

### GC Content Estimation

GC content for sequences matching conserved miRNAs (supplementary table S1, Supplementary Material online) was calculated and sequences were sorted into incremental bins of GC%, at 0.1% increments. Since multiple forms of each miRNA exist and were identified, the GC content for any given miRNA species within and between samples is not constant. Therefore, sequence identities of the three differentially expressed miRNAs miR159, miR319, and miR396 were then identified, and the bins containing the majority of those sequences for each sample were designated as the “GC location” for the relevant miRNA (supplementary fig. S6 and table S3, Supplementary Material online).

### Half-Life Estimation

To estimate the half-life of RNA at the site, we used a per-seed calculation from modern and archeological barleys since per-mass calculations would be skewed due to desiccation. We observed a mean RNA value of 4.45 μg per seed in the archeological, and 65.2 μg per seed in the modern sample of the closest geographical match (Giza). We then calculated the RNA half-life (*T*_1/2_) using the equation:

### T_1/2_  = (T x log2)/(Log (V_1_/V_2_))

where *T* is the elapsed time, *V*_1_ is the amount of modern RNA in μg, and *V*_2_ is the amount of archeological RNA in μg. We used archeological dates of the Late Christian sample of 900–600 years BP to calculate the half-life estimation of 155–232 years.

### Statistical Methods

**Fig. 1.**
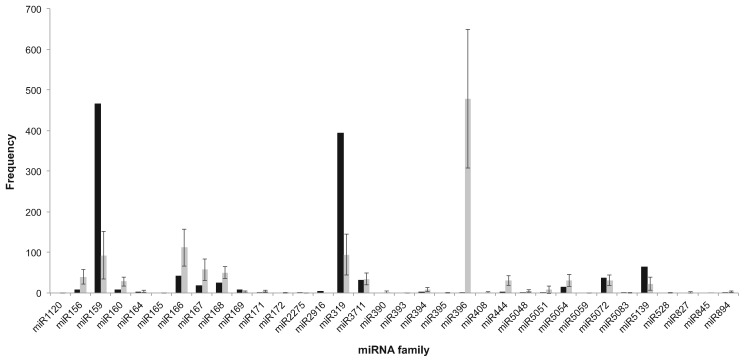
Comparison of conserved miRNA levels in archeological and modern barleys. Frequencies of the modern barleys have been normalized and collated into a single bar for display purposes. The black series represents the archeological sample and the grey series represents the controls. Error bars represent standard deviation between control samples.

Comparability of miRNA frequencies between control samples was confirmed using a correlation regression matrix in GraphPad Prism (version 6.1 for Mac, GraphPad Software, La Jolla California USA). Frequencies of conserved miRNA families were normalized between the control samples by dividing the frequency of each miRNA family in each sample by the ratio of total miRNA frequency to total reads in that sample, and then multiplying that value by the mean ratio of total miRNA frequencies to total reads (0.00165). The adjusted values were then used to normalize the miRNA frequencies of the archeological sample, by calculating the mean adjusted total miRNA frequency per control (1145.5) and dividing this by the total miRNA frequency of the archeological sample to give an adjustment ratio of 0.59. Raw frequencies of miRNA families in the archeological sample were multiplied by this ratio to give an adjusted frequency. Comparisons of modern and archeological were simplified for graphical representation by calculating the normalized mean of all control samples for each miRNA family and plotting these values against normalized archeological reads for each miRNA family. Standard deviations between controls were expressed as error bars (fig. 1).

Likelihood kernels were generated using a standard Poisson process for figures S4 and S5 where low copy numbers occurred (<50). Large RNA count numbers are known to deviate from pure Poisson processes (Hebenstreit etal. 2011), but extended Poisson processes are robust for describing counts (Faddy 1997). In the case of miRNA populations we estimated the ratio of variance to mean by taking point estimates of mean, variance and ratio (*r*) of normalized miRNA counts for each miRNA, and obtained the average value. The likelihood kernel was then based on a Normal approximation of an extended Poisson process using a variance equal to the product of the mean and the ratio (*r*), using equation 5.1a.a of Leonard and Hsu (2001). The likelihoods show the relative likelihood for the underlying mean count number giving rise to the observed counts in each case. In the case of GaMyb, PCF5, and PCF6 fragments, we corrected frequencies within the controls by taking the arithmetic mean of matching fragments across all samples and then calculating the mean ratio from the relative endogenous ratios across all controls. This was done to compensate for low-frequency fragments being over- or under-represented considering an average individual correction factor of 279 between modern and ancient, this factor stemming from the level of data generated (controls were 19-plexed on MiSeq vs. 1-plex HiSeq 2000 for the archeological).

## Supplementary Material

Supplementary data are available at *Molecular Biology and Evolution* online.

## Supplementary Material

Supplementary Data
