# Supplementary material for: Small RNA Activity in Archeological Barley Shows Novel Germination Inhibition in Response to Environment
Source: Mol Biol Evol. 2017 Jun 24;34(10):2555–62. doi: 10.1093/molbev/msx175 (PMC5850308; doi:10.1093/molbev/msx175)
Supplement: Supplementary Data [file msx175_supp.pdf]

Figures and tables

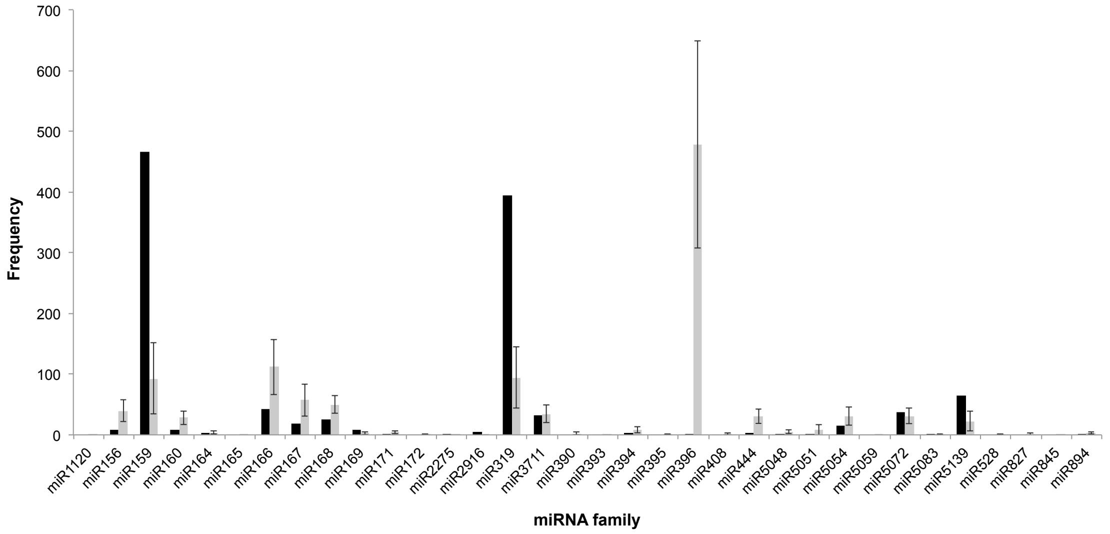

Figure 1: Comparison of conserved miRNA levels in archaeological and modern barleys. Frequencies of the modern barleys have been normalized and collated into a single bar for display purposes. The black series represents the archaeological sample and the grey series represents the controls. Error bars represent standard deviation between control samples.

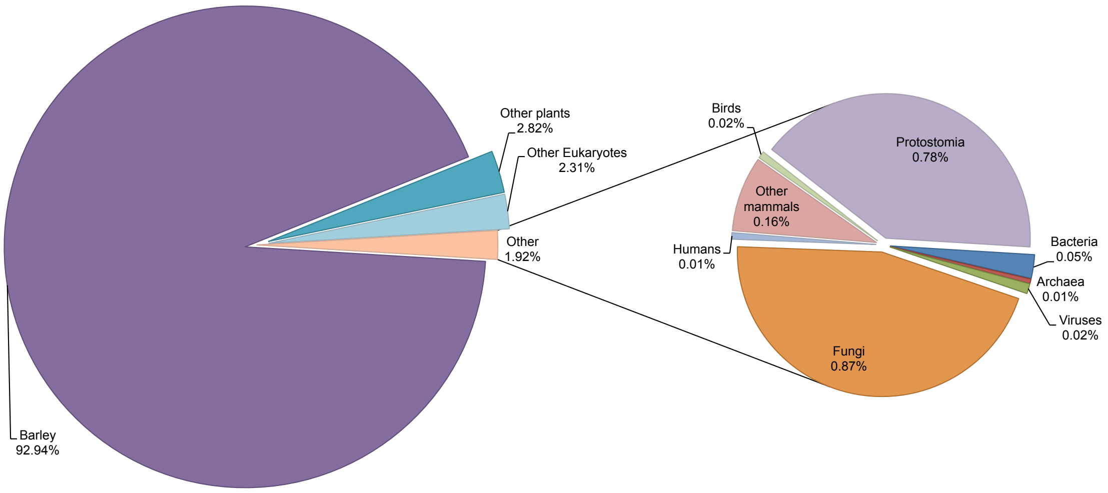

Figure S1: Metagenomic breakdown of Illumina reads from the archaeological barley. The unusually low level of contamination in this sample suggests the authenticity of the Illumina data generated from it.

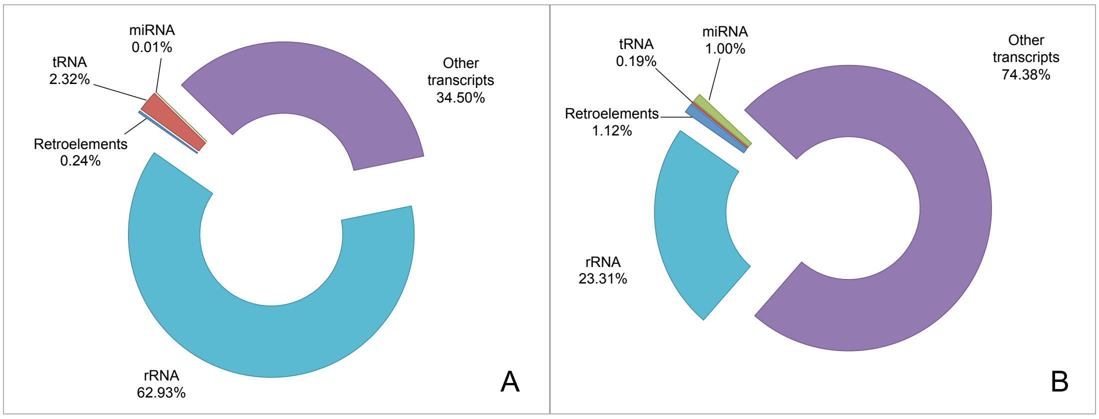

Figure S2: Breakdown of RNA classes. Panel A: Archaeological, actual values. Panel B: modern samples, mean values.

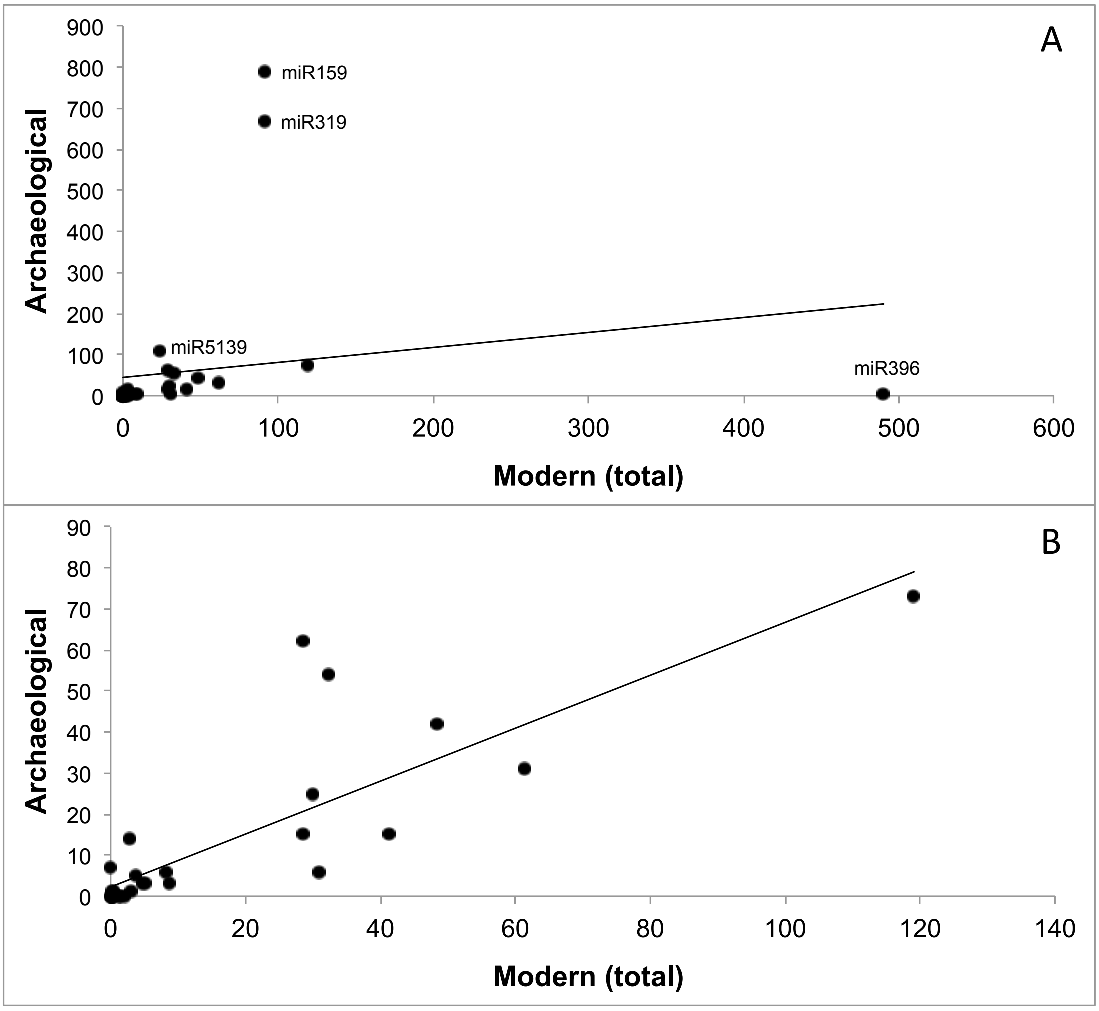

Figure S3: Linear regressions of modern and archaeological barleys. Panel A, including miR159, miR319 and miR396. Panel B; increased correlation of background miRNA species following removal of major functional species.

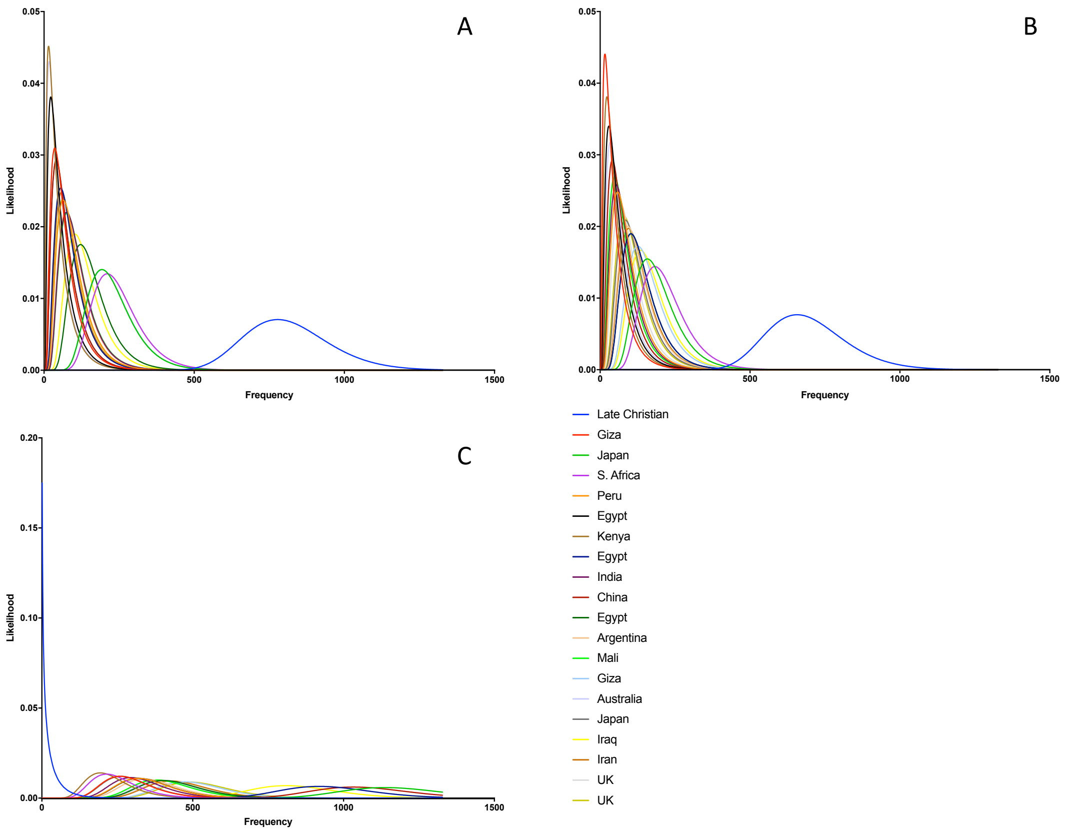

Figure S4: Posterior Poisson distributions of three differentially expressed microRNAs across archaeological (blue) and modern (others) barleys. Panel A: miR159. Panel B: miR319. Panel C: miR396.

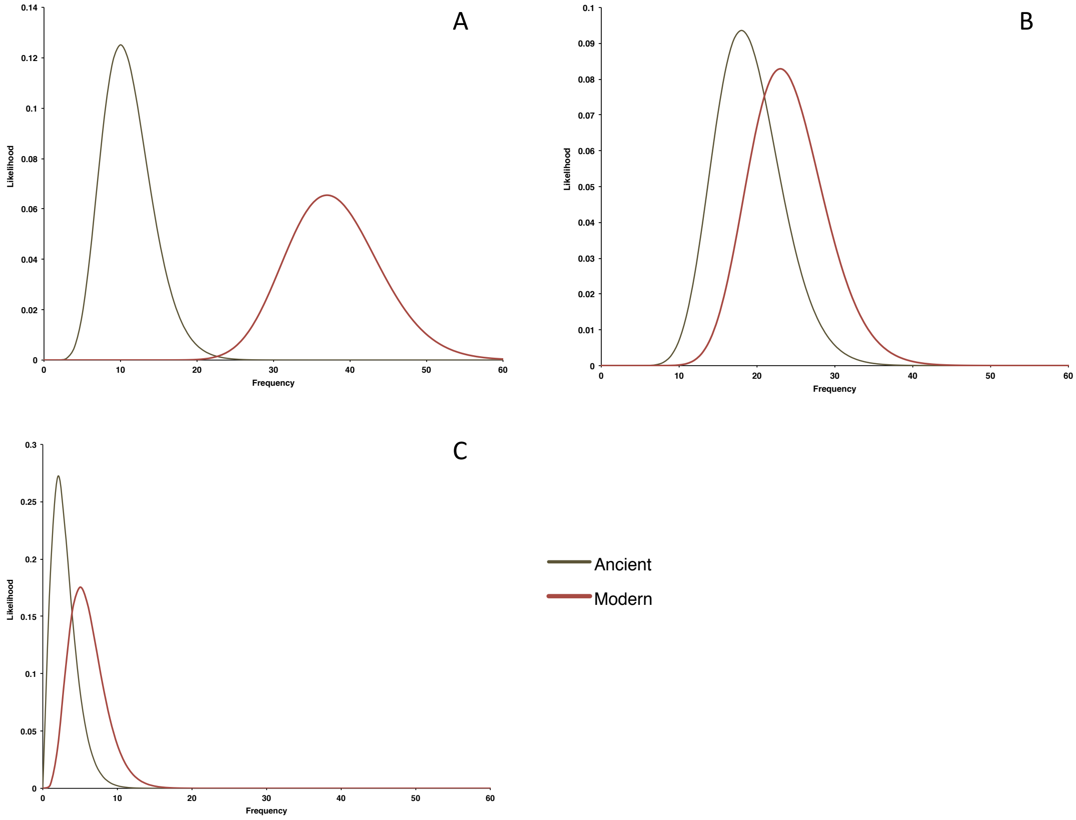

Figure S5: Posterior Poisson distribution of class II TCP domain-containing proteins targeted by miR396. Panel A: GAMYB. Panel B: PCF5. Panel C: PCF6.

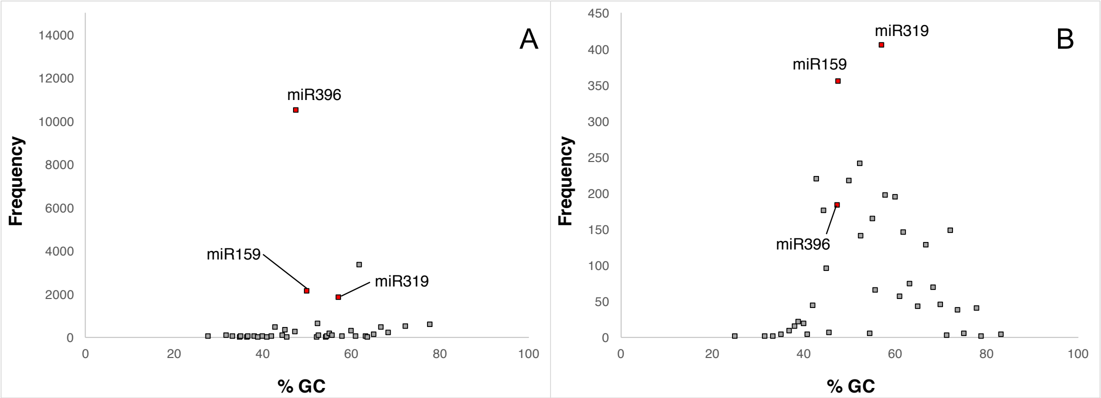

Figure S6: Frequency of miRNA reads according to GC content. GC content for all conserved microRNA reads was calculated and placed into bins of 0.1%. The three most differentially expressed miRNA species (miR159, miR319 and miR396) are highlighted as red data points, placed where the average GC content of the reads identified as such falls into that bin. We note a weak positive correlation between frequency and GC content for all samples. Panel A: summed modern values. Panel B: archaeological values.

	Archaeological	Modern control samples																		
miRNA Family	1 Late Chrisitan	1 Giza	2 Japan	3 S. Africa	4 Peru	5 Egypt	6 Kenya	7 Egypt	8 India	9 China	10 Egypt	11 Argentina	12 Mali	13 Giza	14 Australia	15 Japan	16 Iraq	17 Iran	18 UK	19 UK
miR1120	0	0	2	0	0	0	0	0	0	0	0	0	0	1	0	0	0	0	0	0
miR156	15	2	144	36	28	13	23	35	42	57	29	38	67	57	38	29	56	21	37	31
miR159	790	46	204	222	75	32	24	66	86	51	133	84	87	205	26	51	114	69	88	74
miR160	15	8	55	1	29	23	21	16	48	39	44	22	29	36	18	24	30	29	21	50
miR164	5	0	10	1	3	3	5	2	5	8	11	4	3	3	2	1	2	1	6	3
miR165	0	0	0	0	0	1	0	0	0	0	0	0	0	1	0	0	0	0	0	0
miR166	73	22	445	133	75	66	40	100	151	144	133	118	142	183	63	77	100	81	77	113
miR167	31	12	264	34	35	40	50	64	56	69	47	61	82	86	34	58	67	45	26	36
miR168	42	29	64	38	43	38	25	23	60	68	60	41	52	96	35	48	52	41	46	60
miR169	14	1	2	0	3	1	1	2	0	4	10	5	1	8	5	1	2	3	3	3
miR171	3	1	4	1	5	5	6	1	9	4	9	3	11	8	3	4	1	4	4	4
miR172	0	0	0	0	1	0	1	0	0	1	1	0	0	0	0	0	0	0	1	0
miR2275	1	0	1	0	0	0	0	0	0	0	0	0	0	0	0	1	0	0	0	0
miR2916	7	0	0	0	0	0	0	0	0	0	0	0	0	0	0	0	0	0	0	0
miR319	669	25	169	194	69	39	32	114	64	51	114	103	59	137	44	95	146	106	77	90
miR3711	54	22	22	4	41	38	36	36	28	32	39	38	35	31	24	12	52	53	29	44
miR390	0	0	4	1	0	3	5	1	5	0	3	1	2	8	0	1	2	2	2	1
miR393	0	0	0	0	0	1	0	0	1	0	0	0	0	0	0	0	0	1	0	0
miR394	6	2	19	0	8	8	7	8	8	7	15	6	6	16	0	11	6	6	11	13
miR395	0	0	0	0	0	1	0	0	0	1	1	1	1	0	0	0	0	0	3	0
miR396	3	271	1149	226	330	332	205	931	307	1045	415	350	391	485	407	274	822	441	431	502
miR408	0	2	0	0	0	1	2	1	4	4	1	3	1	1	1	0	1	3	2	0
miR444	6	14	61	5	30	26	18	28	19	33	53	31	40	63	16	16	31	30	28	47
miR5048	3	4	25	2	5	3	5	4	1	4	5	6	2	8	5	5	4	4	3	3
miR5051	3	4	71	12	5	2	4	18	1	3	4	3	5	3	2	12	4	1	6	6
miR5054	25	14	28	4	55	40	36	44	23	34	35	35	33	34	28	12	38	34	7	35
miR5059	0	0	0	0	0	0	0	0	0	0	1	0	1	0	0	0	0	0	0	0
miR5072	62	37	11	23	38	19	34	20	33	45	34	37	27	46	26	21	33	17	15	28
miR5083	1	0	0	0	0	0	0	0	0	2	1	0	0	0	2	0	0	1	0	0
miR5139	109	13	61	37	19	9	10	15	24	112	18	23	13	33	32	15	6	5	7	6
miR528	0	2	0	0	0	0	0	2	0	0	0	0	1	0	0	0	0	0	0	0
miR827	0	2	0	0	0	3	4	2	1	2	1	1	2	2	0	0	0	3	4	0
miR845	0	0	0	0	0	0	0	0	0	0	1	0	0	0	0	0	0	0	0	0
miR894	1	3	3	2	4	0	1	7	2	0	1	3	4	7	3	0	4	2	2	8
**Total**	1938	536	2818	976	901	747	595	1540	978	1820	1219	1017	1097	1558	814	768	1573	1003	936	1157
**Total reads for each**	Not applicable	541759	941447	741158	696256	490405	454368	756179	538418	644253	640912	684441	579008	790111	764536	656373	996102	675880	893175	742469
**Ratio miRNA of total**	Not applicable	0.00098937	0.002993265	0.001316858	0.001294064	0.001523231	0.001309511	0.002036555	0.001816433	0.002824977	0.001901977	0.001485884	0.00189462	0.001971875	0.001064698	0.001170066	0.001579156	0.001483991	0.001047947	0.001558314

Table S1: Raw read counts of conserved miRNA in modern and archaeological barleys. Ratios of miRNA to total read number are shown as basis for correction between miRNA families in the modern samples. The corrected values are then used to calculate a second correction factor to compare all modern samples against the archaeological sample (adjusted data not shown).

		Archaeological	Modern control samples																		
	**Sample**	1 Late Chrisitan	1 Giza	2 Japan	3 S. Africa	4 Peru	5 Egypt	6 Kenya	7 Egypt	8 India	9 China	10 Egypt	11 Argentina	12 Mali	13 Giza	14 Australia	15 Japan	16 Iraq	17 Iran	18 UK	19 UK
	**Total (18-25 size)**	24011053	39833	195508	133217	110352	71135	64618	151656	77360	107564	100533	138556	105123	156101	145498	109559	157275	113248	132951	135439
**mRNA**	**mRNA (total)**	8302629	25165	139866	95505	70634	45782	39034	113210	47777	72444	65973	96286	66865	108701	110573	84108	108965	67054	70232	74764
	**Retroelements**	57650	529	1706	642	1671	1197	1147	1801	1246	1451	1389	1256	1362	1382	879	768	1389	1141	953	1404
	**Other mRNA**	8244979	24636	138160	94863	68963	44585	37887	111409	46531	70993	64584	95030	65503	107319	109694	83340	107576	65913	69279	73360
	**Proportion retroelements**	0.69%	2.10%	1.22%	0.67%	2.37%	2.61%	2.94%	1.59%	2.61%	2.00%	2.11%	1.30%	2.04%	1.27%	0.79%	0.91%	1.27%	1.70%	1.36%	1.88%
	**rRNA**	15110652	9287	46381	36665	23619	14866	12234	38030	15311	24759	22163	35787	22028	40570	47197	35940	41023	21944	22701	22601

Table S2: Raw counts of reads assigned as specific RNA types in archaeological and modern barleys.

Modern		Archaeological	
% GC	Freqency	% GC	Freqency
27.8	6	25.0	1
31.8	73	31.6	1
33.3	9	33.3	1
34.8	2	35.0	3
35.0	14	36.8	8
36.4	4	38.1	14
36.8	7	38.9	21
38.1	40	40.0	18
38.9	2	40.9	3
40.0	17	42.1	43
40.9	3	42.9	219
42.1	6	44.4	175
42.9	424	45.0	95
44.4	47	45.5	6
45.0	319	47.4	183
45.5	3	47.6	354
47.4	238	50.0	216
47.6	10470	52.4	241
50.0	2134	52.6	140
52.2	1	54.5	4
52.4	598	55.0	164
52.6	70	55.6	65
54.2	1	57.1	405
54.5	6	57.9	197
55.0	158	60.0	194
55.6	49	61.1	56
57.1	1834	61.9	145
57.9	33	63.2	73
60.0	287	65.0	42
61.1	39	66.7	128
61.9	3335	68.4	68
63.2	37	70.0	45
63.6	2	71.4	2
65.0	105	72.2	148
66.7	444	73.7	37
68.4	191	75.0	4
72.2	505	77.8	40
77.8	569	78.9	1
		83.3	3

Table S3: GC content of identified miRNA, after figure S6. Sequences were ordered into bins of GC content at 0.1% increments. Red shading indicates GC bin of the majority of miR396-associated reads, blue indicates miR159-associated reads, and green indicates miR319-associated reads. GC bins are calculated based on miRNA matches within the sample; since multiple forms of each miRNA exist, the exact GC content of a single designation (e.g. miR159) varies between samples. For example, while only 3 miR396 sequences were identified in the archaeological sample, their mean GC content clusters with others, hence their clustering with 180 other sequences.
